# Evaluation of Ki-67 as a Prognostic Marker in Diffuse Large B-Cell Lymphoma—A Single-Center Retrospective Cohort Study

**DOI:** 10.3390/curroncol28060383

**Published:** 2021-11-08

**Authors:** Fabian Huber, Elisabeth Zwickl-Traxler, Martin Pecherstorfer, Josef Singer

**Affiliations:** 1 Karl Landsteiner University of Health Sciences, 3500 Krems, Austria; 01326669@edu.kl.ac.at (F.H.); martin.pecherstorfer@krems.lknoe.at (M.P.); 2 Department of Internal Medicine 2, University Hospital Krems, 3500 Krems, Austria; elisabeth.zwickl-traxler@krems.lknoe.at

**Keywords:** diffuse large B-cell lymphoma, DLBCL, Ki-67, survival, international prognostic index, treatment response

## Abstract

Background: Diffuse large B-cell lymphoma (DLBCL) is the most common non-Hodgkin lymphoma and prognostic information is essential in finding the right treatment. This study evaluated the prognostic significance of Ki-67 in patients with DLBCL. Methods: Patients with DLBCL, treated with first-line R-CHOP, were retrospectively analyzed in groups of high (>70%) and low (≤70%) Ki-67. Parameters of interest were the international prognostic index (IPI), treatment response, progression-free survival (PFS) and overall survival (OS). A chi-squared test or Fisher’s exact test was conducted to analyze categorical variables. Kaplan–Meier and log-rank tests were applied for survival analyses. Finally, a multivariate linear regression analysis was performed, including gender, Ki-67 ≤ 70% or >70%, IPI and presence of B symptoms. Results: Overall, 58 patients were included. No significant association was found between Ki-67 status and IPI (*p* = 0.148) or treatment response (*p* = 0.373). Survival in patients with high Ki-67 was significantly inferior with respect to OS (*p* = 0.047) but not PFS (*p* = 0.138). Multivariate linear regression, however, yielded only IPI as a risk factor for OS. Conclusion: Future studies with larger patient cohorts are needed in order to elucidate the prognostic role of Ki-67 in patients with DLBCL treated with R-CHOP.

## 1. Introduction

Diffuse large B-cell lymphoma (DLBCL) accounts for approximately 25–35% of non-Hodgkin lymphomas (NHL) [1] and, despite continuous research for new and better therapies, R-CHOP is still considered the standard first-line therapy [2,3]. Ki-67 is a marker of cellular proliferation expressed as percentage of cells positively stained. Adding to the heterogeneity inherent in DLBCL, the literature on the prognostic value of Ki-67 is inconsistent [4]. In DLBCL, some studies found high Ki-67 expression to be associated with inferior event-free survival (EFS), progression-free survival (PFS) and overall survival (OS) [5,6,7,8], as well as a higher international prognostic index (IPI) [9]. Other studies could not confirm these associations [10,11,12]. Hasselblom et al. even showed low rather than high Ki-67 status to be an adverse prognostic factor [13]. Moreover, it was claimed that the prognostic value of Ki-67 expression is limited and rather dependent on other patient features, such as bulk and IPI [14] or age [15]. It was also found that high Ki-67 expression is more common in non-germinal-center B-cell (non-GCB)-like DLBCL and is associated with c-MYC expression, which might partly explain the poorer outcome in those patients [16]. However, studies addressing Ki-67, IPI and treatment responses are not well established, as described in a systematic meta-analysis [4] which found that there is no clear relationship with IPI and that there are too few studies concerning treatment outcomes. Indeed, findings so far have been conflicting [6,7,8]. The present single-center retrospective cohort study thus aims to evaluate the prognostic significance of Ki-67 in patients treated with R-CHOP on progression-free survival (PFS) and overall survival (OS). Moreover, differences with respect to the IPI and treatment responses were analyzed.

## 2. Materials and Methods

### 2.1. Patients

This retrospective single-center cohort study evaluated patients with DLBCL, NOS, at the University Hospital Krems over a time period from January 2008 to December 2017. Data were acquired from the hospital’s data store of patients’ records. 

Inclusion criteria were a newly diagnosed DLBCL and first-line treatment with R-CHOP, with or without radiotherapy. In addition, all cycles must have been carried out at the University Hospital Krems. 

Patients were excluded if they had any diagnosis other than DLBCL and if they had any history of tumors or transformation from previously diagnosed indolent lymphoma that had progressed or mutated to DLBCL (e.g., Richter’s transformation). An exception was made for previously healthy patients who had two histologic entities (e.g., follicular lymphoma and DLBCL) within the same histological specimen upon initial histopathologic evaluation. In those cases, DLBCL was considered the determining entity. Any patient who had been treated at another hospital at any point in time was excluded. Patients were also ineligible if they had had neither CT nor PET-CT scans for response evaluation, except for patients who were in such a deteriorated health condition upon induction therapy that they died before either treatment could be finished or response assessment could be carried out. These cases remained in the study and were judged as progressive disease. Finally, patients were excluded from the respective analyses if critical parameters could not be found. 

### 2.2. Histopathologic Evaluation

The Ki-67 (30-9)-Rabbit Monoclonal Primary Antibody (Ventana) was used for evaluation. Ki-67 reactivity was estimated pre-therapy using 20× or 40× objective magnification. The reactivity for Ki-67 was assessed by estimating the proportion of positive versus negative nuclei; the numbers provided represent the percentage of reactive nuclei. The calculation did not vary over time as it was performed consistently by the in-house pathologist or the same expert reference hematopathologist. An image analysis system was not used. 

### 2.3. Parameters

The objective was to evaluate patients for differences based on immunohistochemically determined Ki-67 values at a cut-off of 70%. Within these groups of high (>70%) and low (≤70%) Ki-67, the parameters of interest were survival (i.e., PFS and OS), treatment response (i.e., complete response (CR), partial response (PR), stable disease (SD), progressive disease (PD)) and international prognostic index (IPI). 

If the IPI was not readily available from patient records, the relevant factors for its calculation were searched for, and if available, the risk group was determined manually. The stage and response were derived from patients’ records. If not explicitly mentioned, patients were assessed according to the Lugano classification. Patients were evaluated for maximally achieved response. If there was unclear or minimal residual disease in radiology records without a clear statement on CR or PR, patients were classified as CR if they were symptom-free and/or no further therapy was applied over the entire course of follow-up. In cases where the residual disease and inflammatory changes could not be distinguished, the decision on response was based on whether further clinical examination or biopsy confirmed malignant tissue.

Survival parameters were chosen as recommended by the National Cancer Institute (cancer.gov, accessed on 25 October 2021): PFS was defined as the time period from treatment initiation to either progression, regrowth, last follow-up or death. OS was defined as the time from treatment initiation to either the time of death or, if remission persisted, to last follow-up irrespective of relapse or progressive disease. Survival times were calculated in months and were rounded to half a month.

### 2.4. Statistical Analyses

Survival was assessed by means of a Kaplan–Meier analysis and compared using a log-rank test. As data on IPI and response were grouped data and Ki-67 was also defined as a categorical variable, analysis of these parameters was carried out using Pearson’s chi-squared test or Fisher’s exact test. A two-sided significance level of *p* < 0.05 was determined. Demographic data were evaluated for differences using chi-squared or Fisher’s exact tests and a *t*-test or Mann–Whitney *U* test, depending on the normal distribution as assessed by the Shapiro-Wilk test. There was no stratification with regard to variables such as age, sex, BMI or comorbidities.

To further investigate factors associated with overall survival, a multivariate linear regression analysis was performed, including gender, Ki-67 ≤ 70% or >70%, IPI and presence of B symptoms. R-squared and ANOVA tests were used to assess the adequacy of the models. Multicollinearity was assessed with variance inflation factors to confirm the independence of variables included in the regression model. The final analyses were conducted using IBM SPSS Statistics (versions 26.0.0.0 and 27.0.1.0; Armonk, NY, USA).

## 3. Results

### 3.1. Patient Population

Overall, 58 patients were included in the study. The complete screening algorithm for inclusion and exclusion in this study can be found in Figure S1. Patient characteristics can be seen in Table 1. The only significant difference with regard to the demographic variables was found for bulky disease. In this study, patients with low Ki-67 had proportionally more cases of bulk compared to those with high Ki-67 (*p* = 0.028). The median Ki-67 was 90%. There were 46 and 12 patients in the high and low Ki-67 cohorts, respectively. Loss to follow-up (i.e., last visit longer than one year prior to the final date of data acquisition) occurred in eight patients, who remained in the final survival analyses until their respective last visits. Of those, two patients were in the low Ki-67 group and six patients were in the high Ki-67 group. All but one of those patients achieved CR (the other being PR); one had additional radiation therapy.

#### 3.1.1. Ki-67 and IPI

50 patients were analyzed for Ki-67 and IPI. Eight patients were excluded due to missing information (all in the high Ki-67 group). In two patients, information on ECOG was missing but the overall IPI score was available from the records. Though a tendency towards high-risk categories in the high Ki-67 group could be argued, based on Table 2, there was no significant difference in our study population (Fisher’s exact test, *p* = 0.148).

#### 3.1.2. Ki-67 and Response

All 58 patients were evaluable for treatment response. As can be seen in Table 2, all patients with low Ki-67 achieved CR after R-CHOP treatment, whereas in the high Ki-67 cohort responses differed greatly. However, based on our data, no significant difference between these two groups was found (Fisher’s exact test, *p* = 0.373).

#### 3.1.3. Ki-67 and Survival

**Figure 1:**
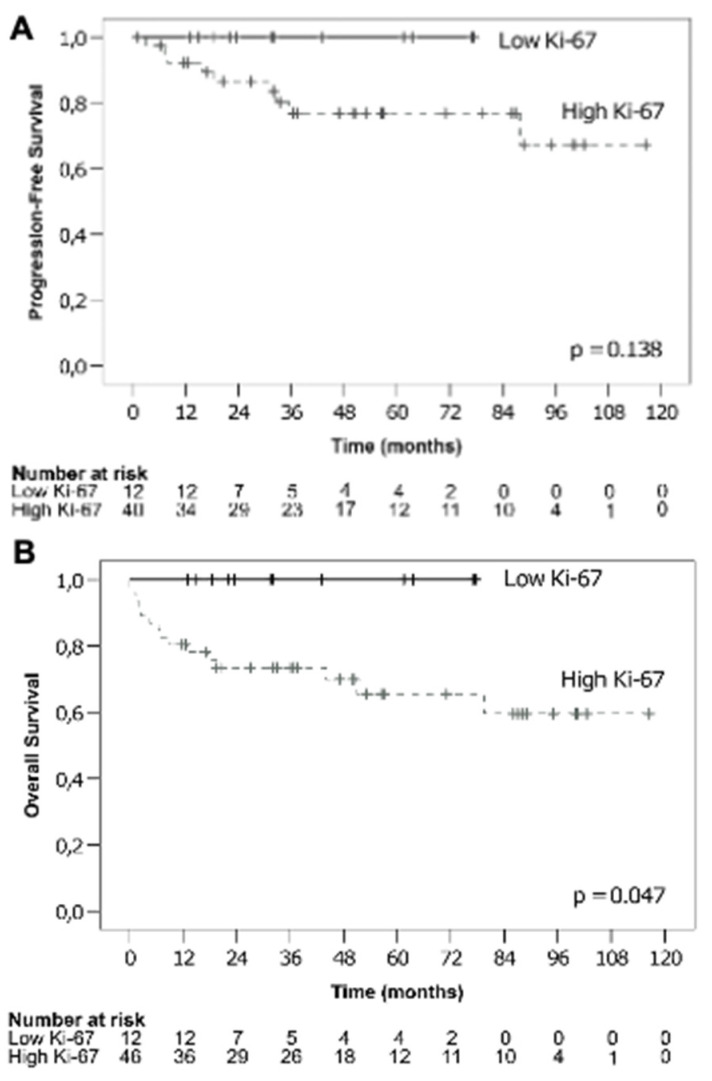
Kaplan–Meier analysis comparing survival rates according to low (continuous line) and high (dashed line) Ki-67. Differences in progression-free survival (PFS) did not reach statistical significance (**A**) and high Ki-67 was associated with worse overall survival (OS) (**B**).

A total of 52 patients were eligible for evaluation of PFS. Of these, 12 patients belonged to the low Ki-67 group and 40 to the high Ki-67 group. There was a tendency towards inferior PFS in patients with high Ki-67 DLBCL, as depicted in Figure 1A. This difference, however, was not significant (χ^2^ = 2.202, *p* = 0.138). The estimated PFS for low Ki-67 was 100% at 1, 2 and 3 years. In the high Ki-67 group, PFS at 1, 2 and 3 years was 92.2%, 86.5% and 76.9%, respectively.

All 58 patients were eligible for OS analysis. As can be seen in Figure 1B, the survival analysis revealed significantly inferior OS in patients with high Ki-67 expression compared to those with low Ki-67 (χ^2^ = 3.946, *p* = 0.047). The high Ki-67 group had an estimated OS at 1 year of 80.4%, while in the low Ki-67 group no events took place, resulting in a 1-year OS of 100%. This was the time point at which the status of almost all patients was known, except for one patient in the high Ki-67 group for whom data were censored at 11.5 months. Thus, this factor represents the most accurate estimation. After 2 years and at 3 years, the OS in patients with high Ki-67 was estimated at 73.4%. In patients with low Ki-67, no events occurred over the entire follow up period resulting in an OS of 100% at 2 years and 3 years. 

In general, the percentages given above are estimates, as there were censored cases already within the first year. Overall, patients with low Ki-67 expression were monitored for a shorter period of time due to the fact that in this cohort a higher proportion of patients were treated in later years of the recruitment period. Unfortunately, no statistics on mean or median survival could be calculated because neither group displayed enough events to reach below the 50% threshold of cumulative survival in the Kaplan–Meier analyses.

### 3.2. Multivariate Analysis

To identify further factors associated with overall survival, a multivariate linear regression analysis was performed, including gender, Ki-67 ≤ 70% or > 70%, IPI and the presence of B symptoms. As displayed in Table 3, this analysis yielded only IPI as a significant risk factor.

## 4. Discussion

This single-center retrospective cohort study investigated whether the expression of Ki-67 could be prognostic when categorized into groups of ≤70% (low Ki-67) and >70% (high Ki-67) in DLBCL patients undergoing first-line therapy with R-CHOP.

The analysis of IPI and Ki-67 expression in this study revealed no significant differences between low and high Ki-67 expression, although judging from the distribution of risk groups shown in the previous section one might observe a tendency towards higher risk in the high Ki-67 patient cohort. This finding is supported by other studies that evaluated Ki-67 in patients with DLBCL. Broyde et al. [14] conducted a similar study with a Ki-67 cut-off value at 70% in a broader lymphoma population and found that, in the subgroup of DLBCL, the IPI score did not significantly correlate with the Ki-67 index, although it did show an association with ECOG performance status. Ott et al. [12], who investigated patients from the prospective RICOVER-60 trial receiving CHOP with or without rituximab, also could not find a correlation. A meta-analysis on the prognostic value of Ki-67 performed by He et al. [4] found that, as of 2014, there was no correlation of Ki-67 with IPI. Thus, despite differences in the statistical tests applied, the results from the present study can be considered consistent with these findings.

We also analyzed the potential relationship between treatment response and Ki-67 expression. Again, no significant differences were observed between the two groups. Yoon et al. [8] found no significant difference in the CR rate, although they observed a higher relapse rate after CR in high Ki-67 patients, while Li et al. [6] and Gaudio et al. [7] proposed a significant influence of Ki-67 on response rate. Several explanations might account for our finding. On the one hand, the definition of CR was partly based on clinical judgment as described above. On the other hand, most patients in fact achieved CR, so that other response categories were proportionally underrepresented. This underlines the remarkable effect of immuno-chemotherapy with rituximab and CHOP. 

With regard to survival parameters, the present study did not display significant differences in PFS between the groups with low and high Ki-67 expression. Upon close inspection of the Kaplan–Meier curves, our results may hint at an inferior outcome with a higher relapse rate in the high Ki-67 cohort. Comparing this finding with previous studies, Gaudio et al. [7] reported a significant relationship between high Ki-67 and worse PFS that was independent of IPI status. In addition, Song et al. [5] found worse PFS in patients irrespective of bone marrow involvement. Li et al. [6] stated that PFS was worse in patients with high Ki-67 expression, but here, this prognostic factor was dependent on IPI, being only significant at IPI ≥ 2. It must be stated that the definition of PFS in the study by Li et al. was different from that used in our study, as there, PFS was calculated from the time of initial diagnosis. With this definition, Li et al. [6] reported a 3-year PFS in the high Ki-67 cohort of 56.4%, compared to 73.3% in the low Ki-67 expressing patient group. 

In contrast to PFS, OS did reach statistical significance with inferior outcomes in patients with high Ki-67 compared to low Ki-67 expression. This was not the case when tested further using a multivariate linear regression analysis. Here, only IPI remained as a significant risk factor.

In the literature, several studies also investigated the overall prognostic value of Ki-67 for OS [4,5,6,7,8,14,15,17]. In detail, Gaudio et al. [7] argued that high Ki-67 status remained a prognostic factor, using a multivariate analysis, and that it was also independent of IPI. In contrast, Koh et al. [15] reported that Ki-67 was only prognostic when analyzed in late-elderly patients and non-GCB DLBCL, while Broyde et al. [14] found Ki-67 to be of added prognostic significance only in patients with IPI ≤ 2. In addition, Song et al. [5] stated that high Ki-67 adds to a worse prognosis in patients with bone marrow involvement. Yoon et al. [8] found that high Ki-67 expression was a significant prognostic factor only in DLBCL, NOS, while in the overall group it did not reach prognostic significance. Other studies found no association of Ki-67 with OS [10,11,12]. It has been argued that some of these conflicting results might be attributed to analyses conducted prior to the R-CHOP era [6]. Indeed, Salles et al. [17] found that Ki-67 has prognostic relevance in patients treated with R-CHOP but not with CHOP. This assumption is supported by the meta-analysis of He et al. [4], although there was considerable heterogeneity in the Ki-67 cut-off values in the included studies.

Hasselblom et al. [13] postulated that, at a cut-off of 49%, a low rather than a high Ki-67 index is unfavorable with regard to both PFS and OS. However, in addition to the use of CHOP only and a different definition of PFS (from the date of diagnosis), they also did not include patients with abbreviated therapy due to complications and toxicity, all of which might account for this finding. It was suggested by Tang et al. [18] that the combination of BCL2 and Ki-67 might allow for better prognostic information and that a high co-expression indicates a poorer prognosis with regard to PFS and OS compared to patients with low expression; however, this was true only in GCB DLBCL, while in non-GCB DLBCL only PFS was worse. In addition, Ki-67 as a single marker showed worse PFS but not OS in the overall cohort, and only at a cut-off ≥90%, which included proportionately fewer cases compared to patients in the <90% group.

Hence, the issue of whether or not a Ki-67 status of 70% is a good cut-off value should be discussed: on the one hand, it allows for limited prognostic information on survival, but on the other hand, Ki-67 expression below 70% was relatively uncommon in our patient population. We considered whether the median value of Ki-67 would be better suited; however, the median is very much dependent on each individual study population and thus would not be a reliable measure for a broader clinical application. Hence, more investigations may be needed, specifically for the definition of the optimal cut-off value. Optimally, this would include prospective trials with well-defined treatment regimens that do not differ based on Ki-67 status, to avoid the introduction of bias.

There are several limitations to this project. First and foremost, the retrospective character of this study means that it is inevitably subject to unknown confounding factors and is dependent on the quality of the documentation as well as the compliance of the patients. Secondly, this study dealt with data that extended over a long period of time during which medical knowledge has changed. Previous studies might have included other variants of DLBCL and double-/triple-hit lymphoma (a separate diagnostic category in the most recent WHO classification), which was not included in this project. Information on MYC rearrangements especially would be very interesting, as this could be an important confounding factor. Similarly, we could not discriminate in all our cases between GCB- and non-GCB DLBCL due to missing information regarding the early samples in the investigation period. These aspects should be addressed in all further studies on this topic. Thirdly, our sample size was relatively small and there were a number of censored cases. Inhomogeneous entry dates, the calculation of and differences in lengths of follow-up periods and patients that were lost to follow-up might also have distorted the results. Furthermore, the statistics used in the study were not capable of showing, or designed to show, the strength of a correlation. Finally, Ki-67 status was determined by one pathologist only. For future studies, we strongly recommend independent review by another pathologist, to account for interobserver variation. 

## 5. Conclusions

Further large prospective studies are needed to elucidate the prognostic role of Ki-67 in patients with DLBCL treated with R-CHOP.

## Figures and Tables

**Table 1:** Demographic characteristics of the patient cohort.

Patient Characteristics		Ki-67								*p*-Value
		Low (≤70%) (*N* = 12)				High (>70%) (*N* = 46)				
		*N* (%)	Min.	Median	Max.	*N* (%)	Min.	Median	Max.	
Gender	female	6 (50.0%)				17 (37.0%)				0.513
	male	6 (50.0%)				29 (63.0%)				
Age (years)			25	59	82		18	68	88	0.161
BMI			23.1	27.7	32.7		17.9	25.4	42.7	0.146
Ann nmslyyds Arbor	I	1 (8.3%)				8 (17.4%)				0.405
	II	8 (66.7%)				17 (37.0%)				
	III	1 (8.3%)				6 (13.0%)				
	IV	2 (16.7%)				15 (32.6%)				
B symptoms	no	9 (75.0%)				33 (71.7%)				1.000
	yes	3 (25.0%)				13 (28.3%)				
Extranodal sites			0	1	2		0	1	5	0.124
ECOG	0	7 (58.3%)				15 (41.7%)				
	1	3 (25.0%)				10 (27.8%)				0.857
	2	2 (16.7%)				7 (19.4%)				
	3	0 (0.0%)				3 (8.3%)				
	4	0 (0.0%)				1 (2.8%)				
LDH (U/l)			130	216	812		110	270	3108	0.437
Bulk	No	5 (41.7%)				36 (78.3%)				0.028
	Yes	7 (58.3%)				10 (21.7%)				
Maintenance therapy	No	11 (91.7%)				36 (78.3%)				0.429
	Yes	1 (8.3%)				10 (21.7%)				
2nd line	No	12 (100.0%)				40 (87.0%)				0.328
	Yes	0 (0.0%)				6 (13.0%)				
3rd line	No	12 (100.0%)				44 (95.7%)				1.000
	Yes	0 (0.0%)				2 (4.3%)				
Transplant	No	12 (100.0%)				45 (97.8%)				1.000
	Yes	0 (0.0%)				1 (2.2%)				
Radiotherapy	No	7 (58.3%)				32 (69.6%)				0.360
	Initial	5 (41.7%)				11 (23.9%)				
	Relapse	0 (0.0%)				3 (6.5%)				

BMI: Body Mass Index; ECOG: Eastern Cooperative Oncology Group; LDH: lactate dehydrogenase.

**Table 2:** Analysis of differences in international prognostic index (IPI) and treatment response between low and high Ki-67 groups.

		Ki-67		*p*-Value
		Low (≤70%)	High (>70%)	
		*N* (%)	*N* (%)	
IPI Group	low	7 (58.3%)	19 (50.0%)	0.148
	intermediate-low	4 (33.3%)	4 (10.5%)	
	intermediate-high	0 (0.0%)	4 (10.5%)	
	high	1 (8.3%)	11 (28.9%)	
Response	CR	12 (100.0%)	34 (73.9%)	0.373
	PR	0 (0.0%)	5 (10.9%)	
	SD	0 (0.0%)	1 (2.2%)	
	PD	0 (0.0%)	6 (13.0%)	

IPI: international prognostic index; CR: complete response; PR: partial response; SD: stable disease; PD: progressive disease.

**Table 3:** Multivariate analysis of risk factors for overall survival.

Factors	Unstandardized Coefficients Beta	95.0% CI		*p*-Value	Adjusted R^2^	F Value
		Lower Bound	Upper Bound			
Gender	−4.435	−22.681	13.811	0.627	0.057	1.737
Ki-67 ≤ 70% or > 70%	10.788	−10.244	31.820	0.307		
IPI	−6.477	−12.530	−0.424	0.037		
Presence of B symptoms	−5.252	−26.679	16.175	0.624		

IPI: international prognostic index.

## Data Availability

Data remain at the University Hospital Krems. The datasets used and analyzed during the current study are available from the corresponding author on reasonable request.

## Supplementary Materials

The following are available online at https://www.mdpi.com/article/10.3390/curroncol28060383/s1, Figure S1: Screening algorithm of patients’ records for inclusion and exclusion in this study.
