# Supplementary material for: Evaluation of Ki-67 as a Prognostic Marker in Diffuse Large B-Cell Lymphoma—A Single-Center Retrospective Cohort Study
Source: Curr Oncol. 2021 Nov 8;28(6):4521–9. doi: 10.3390/curroncol28060383 (PMC8628729; doi:10.3390/curroncol28060383)
Supplement: Supplementary file 1 [file curroncol-28-00383-s001.zip › curroncol-1445993-supplementary.pdf]

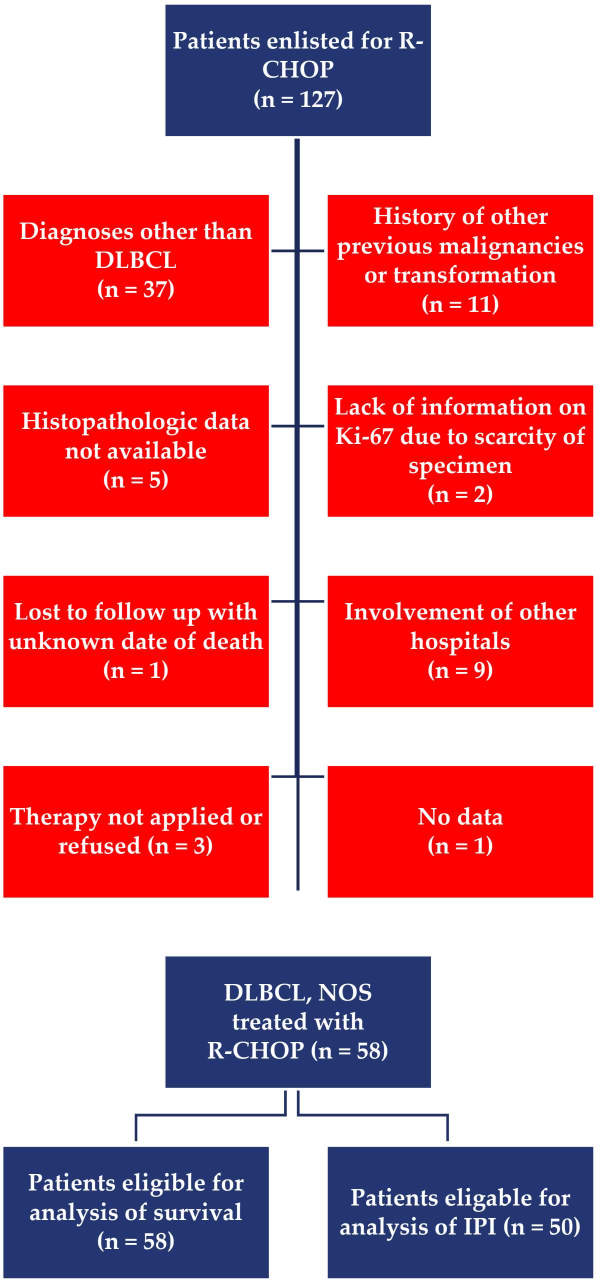

Supplementary Figure S1:

Screening algorithm of patients' records for inclusion and exclusion in this study.
